# Mucosa-associated gut microbiota reflects clinical course of ulcerative colitis

**DOI:** 10.1038/s41598-021-92870-0

**Published:** 2021-07-02

**Authors:** Yuichiro Nishihara, Haruei Ogino, Masaru Tanaka, Eikichi Ihara, Keita Fukaura, Kei Nishioka, Takatoshi Chinen, Yoshimasa Tanaka, Jiro Nakayama, Dongchon Kang, Yoshihiro Ogawa

**Affiliations:** 1 Department of Medicine and Bioregulatory Science, Graduate School of Medical Sciences, Kyushu University, 3-1-1 Maidashi, Higashi-ku, Fukuoka, 812-8582 Japan; 2 Department of Gastroenterology and Metabolism, Graduate School of Medical Sciences, Kyushu University, 3-1-1 Maidashi, Higashi-ku, Fukuoka, 812-8582 Japan; 3 Laboratory of Microbial Technology, Division of Systems Bioengineering, Department of Bioscience and Biotechnology, Faculty of Agriculture, Graduate School, Kyushu University, 744 Motooka, Nishi-ku, Fukuoka, 819-0395 Japan; 4 Department of Clinical Chemistry and Laboratory, Medicine, Graduate School of Medical Sciences, Kyushu University, 3-1-1 Maidashi, Higashi-ku, Fukuoka, 812-8582 Japan

**Keywords:** Ulcerative colitis, Clinical microbiology

## Abstract

This longitudinal study was designed to elucidate whether gut microbiota is associated with relapse and treatment response in ulcerative colitis (UC) patients. Fifty-one patients with UC were enrolled between 2012 and 2017, and followed up through 2020. Colon mucosal biopsy were obtained at enrollment, and 16S ribosomal RNA sequencing was performed using extracted RNA. Of the 51 patients, 24 were in remission and 27 had active UC at enrollment. Of the 24 patients in remission, 17 maintained remission and 7 developed relapse during follow-up. The 7 patients with relapse showed lower diversity, with a lower proportion of Clostridiales (*p* = 0.0043), and a higher proportion of *Bacteroides* (*p* = 0.047) at enrollment than those without relapse. The 27 patients with active UC were classified into response (n = 6), refractory (n = 13), and non-response (n = 8) groups according to their treatment response in 6 months. The refractory and non-response groups showed lower diversity with a lower proportion of *Prevotella* (*p* = 0.048 and 0.043) at enrollment than the response group. This study is the first demonstration that reduced diversity and particular microbes are associated with the later clinical course of relapse events and treatment response in UC.

Inflammatory bowel disease (IBD), including ulcerative colitis (UC) and Crohn’s disease, is characterized by chronic relapsing inflammation of the gastrointestinal tract. Although its exact etiology remains unknown, IBD has been reported to result from an exaggerated T-cell response to environmental factors in a genetically predisposed host^1–3^. The incidence of IBD has rapidly increased worldwide, especially in the Asia–Pacific area; this suggests that alternations of environmental factors, including changes in the composition of the gut microbiota, might play a role in the pathogenesis of IBD^1,2,4^. Studies have shown significant differences in the composition of gut microbiota between patients with IBD and healthy controls^1,3,5^. Notably, reduced diversity of microbiota, characterized by a decrease in *Bacteroides* and an increase in Gammaproteobacteria, has been reported during the active phase of UC^3,5^. However, it remains controversial whether alterations of gut microbiota composition are involved in the pathogenesis of UC.

In clinical practice, it is critical to assess the risk of relapse and to predict long-term treatment response in patients with UC. However, it remains unclear how gut microbiota affect the disease progress in these patients. Thus far, few studies have examined the effects of alteration of gut microbiota composition on the clinical course of IBD, and most studies have been cross-sectional in nature with assessment at a single time point. Moreover, the method of examination of the gut microbiota has been limited. Indeed, the optimal method to analyze the gut microbiota remains unclear. The DNA extracted from fecal samples is usually used to analyze the gut microbiota, but such analysis cannot exclude either dead or dormant microbes, and is easily affected by the stool and mucosal inflammation^6,7^. In contrast, the use of RNA extracts from the gut mucosa can focus on live and active microbes that adhere to the gut mucosa^8–12^.

Thus, this longitudinal study was designed to elucidate the roles of gut microbiota in the pathogenesis of UC through examination of mucosal gut microbiota associations with relapse events and treatment response in patients with UC, by means of RNA-based 16S rRNA analysis.

## Results

### Difference in detection yield of gut microbiota based on combination of samples used and methods applied

We examined whether there was any difference in the detection yield of gut microbiota based on the combinations of samples used and methods applied. In 6 control subjects, we compared the gut microbiota compositions in 4 different conditions: feces DNA-based 16S rRNA, feces RNA-based 16S rRNA, tissue DNA-based 16S rRNA, and tissue RNA-based 16S rRNA. The sequencing coverage was lower with tissue DNA-based 16S rRNA than with the others. The number of observed OTUs was significantly lower and the proportion of unclassified bacteria was significantly higher with tissue DNA-based 16S rRNA than with 3 other conditions (Supplementary Fig. 1A–C). In contrast, the PCoA of β-diversity revealed differences in gut microbiota composition determined by unweighted (PERMANOVA, *p* < 0.0001) and weighted (PERMANOVA, *p* < 0.0001) UniFrac distances between feces and rectal tissues. There were also significant differences in gut microbiota composition determined by unweighted UniFrac distances between tissue DNA-based 16S rRNA and tissue RNA-based 16S rRNA (PERMANOVA, *p* = 0.0001) (Supplementary Fig. 1D). Based on these findings, tissue RNA-based 16S rRNA was used for further analysis.

### Clinical characteristics of patients with UC at enrollment

Table 1 summarizes the clinical characteristics of the patients with UC enrolled in this study. During the follow-up period, 17 of the 24 patients in the endoscopic remission group (MES of 0/1) maintained remission for a median period of 72 months (60–85 months), and 7 of 24 patients developed relapse after a median period of 18 months (16–24 months) after enrollment. Among 7 patients in the relapse group, the median MES at enrollment was 1 (0.5–2), and 4 of 7 patients had an MES of 0. Among 17 patients in the non-relapse group, the median MES at enrollment was also 1 (1–2), and 6 of 17 patients had an MES of 0. No significant difference was observed between the relapse and non-relapse groups. In contrast, among 27 patients in the endoscopically active group (MES of 2/3), the median MES at enrollment in the response, refractory, and non-response groups was 7 (6–9; n = 6), 8 (6–9; n = 13), and 8.5 (6–10; n = 8), respectively. There were no significant differences in these values among the 3 groups. No patients with PSC were enrolled in this study; moreover, no patients enrolled in this study developed colorectal cancer during the follow-up period.

**Table 1:** Clinical Characteristics of Patients with UC at Enrollment.

	UC	MES 0/1	MES 0/1	MES 0/1	*p* value	MES 2/3	MES 2/3	MES 2/3	MES 2/3	*p* value
			Relapse	Non-relapse			Response	Refractory	Non-response	
	Total: n = 51	Total: n = 24	n = 7	n = 17		Total: n = 27	n = 6	n = 13	n = 8	
Female/male	24/27	11/13	6/1	5/12	1	13/14	1/5	8/5	4/4	0.23
Age, yr, median (IQR)	45 (34–58)	43.5 (37–56.8)	42 (37.5–55)	45 (38–56)	0.9	46 (32–59)	44.5 (40–49)	39 (27–67)	47.5 (35–56)	0.99
Disease yr, median (IQR)	6 (3–14)	8 (3–16)	5 (3–12.5)	8.5 (2.8–16.3)	0.79	5 (3–8.5)	5.5 (4.25–7.5)	4 (3–11)	6 (4–10)	0.87
Total Mayo score, median (IQR)	3 (1–8)	1 (0.75–2)	1 (0.5–2)	1 (1–2)	0.74	8 (6–10)	7 (6–9)	8 (6–9)	8.5 (6–10)	0.75
Body mass index, median (IQR)	20.3 (18–23)	19.5(18–22)	18.6 (17–19)	21.0 (18–22)	0.058	20.5 (18–25)	20.5 (20–25)	20.3 (18–25)	21.1 (20–23)	0.39
**Type of disease**					1					0.093
Proctitis	5 (9.8%)	3 (15%)	1	2		2 (7%)	1	0	0	
Left-sided colitis	17 (33.3%)	7 (26%)	2	5		10 (37%)	4	4	2	
Pancolitis	27 (52.9%)	12 (52%)	4	8		15 (56%)	1	9	6	
Right-sided colitis	2 (0.39%)	2 (7%)	0	2		0 (0%)	0	0	0	
**Medication**										
5ASA/SASP	49 (96.1%)	25 (93%)	6	17	0.29	26 (96%)	6	13	7	0.52
Corticosteroids	10 (19.6%)	0 (0%)	0	0	–	10 (37%)	1	5	4	0.45
AZA/6MP	18 (35.3%)	6 (22%)	2	4	1	12 (44%)	1	6	5	0.25
Apheresis	0 (0%)	0 (0%)	0	0	–	0 (0%)	0	0	0	–
Anti-TNFαtherapy	10 (19.6%)	4 (15%)	1	1	0.51	6 (22%)	0	3	3	0.34
Tacrolimus	0 (0%)	0 (0%)	0	0	–	0 (0%)	0	0	0	–
**Duration of remission after enrollment, median (IQR)**			18 (16–24)	72 (60–85)						

*5ASA* 5-aminosalicylic acid, *SASP* salazosulfapyridine, *AZA* azathioprine, *6MP* mercaptopurine, *IQR* interquartile range.

*p* value: Fisher's exact test was used to the categorized data, and Mann-Whiteney U test and One-way ANOVA were used to the continuous data.

### Comparison of gut microbiota composition between rectum and colon

**Figure 1:**
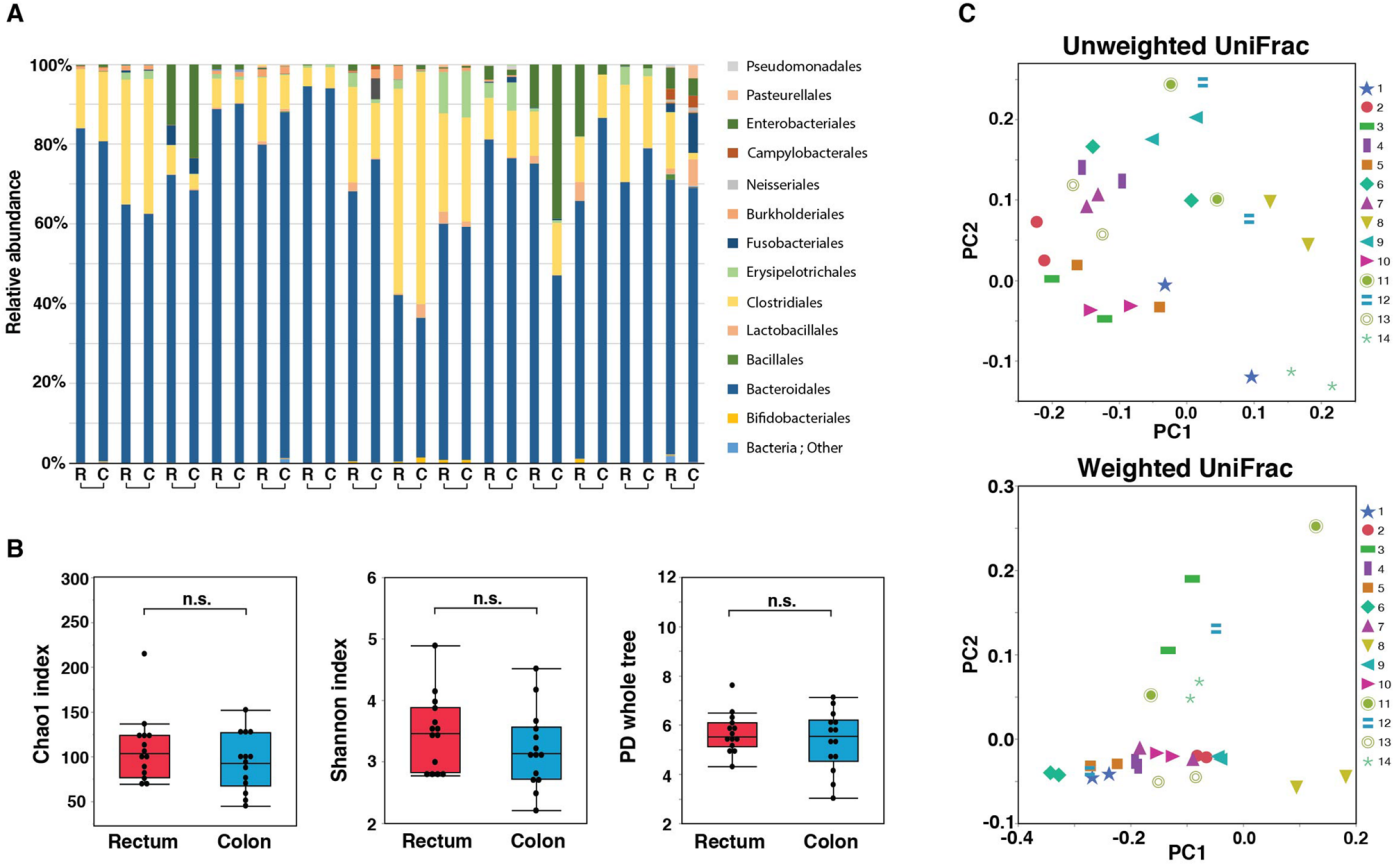
Comparison of gut microbiota composition between rectum and colon in patients with ulcerative colitis (UC). **(A)** Bar graph of the gut microbiota composition of the rectum (R) and colon (C) in the patients with UC at the order level. The brackets underneath indicate one individual. **(B)** Comparisons of α-diversity of gut microbiota composition between rectum and colon as assessed by the Chao1 index, the Shannon index, and whole-tree phylogenetic diversity (PD). **(C)** Principal coordinate analysis of gut microbiota by unweighted and weighted UniFrac between the rectum and colon. Each symbol indicates one patient. n.s., no significant difference between the 2 indicated groups (PERMANOVA, *p* = 1.0).

We investigated whether there was any difference in gut microbiota composition between the rectum and colon among 14 patients who had undergone both rectal and colonic biopsies. These 14 patients comprised 2, 7, 3, and 2 patients with an MES of 0, 1, 2, and 3, respectively. With respect to type of UC, 8 patients had pancolitis, 4 had left-sided UC, and 2 had right-sided UC. The gut microbiota compositions of these 14 patients at the order level are shown in Fig. 1A. There was no significant difference in the α-diversity of gut microbiota composition between the rectum and colon as measured by the Chao1 index (105; 83.1–124 vs. 94.3; 72.8–121), the Shannon index (3.47; 2.88–3.81 vs. 3.14; 2.74–3.50), whole-tree PD (5.52; 5.21–6.00 vs. 5.54; 4.69–6.12), and Pielo’s evenness index (Fig. 1B and Supplementary Fig. 2A). There was also no difference in gut microbiota composition between the rectum and colon as measured by weighted and unweighted UniFrac distances (PERMANOVA, *p* = 1.0) (Fig. 1C). Furthermore, the extent of the weighted interindividual UniFrac distance (rectum-colon; between rectal and colonic samples from the same patients) was significantly lower than the weighted intraindividual UniFrac distance (rectum-rectum; between rectal samples from different patients) (*p* = 0.0008) (Supplementary Fig. 3).

### Comparison of gut microbiota composition between patients with UC and control subjects

We compared gut microbiota composition between 51 patients with UC and 7 control subjects. The Shannon index and Pielo’s evenness index tended to be lower in patients with UC than in controls (*p* = 0.079, *p* = 0.092), while the Chao1 index was significantly lower in patients with UC than in controls (*p* = 0.023) (Supplementary Figs. 2D and 4A). Furthermore, there were significant differences in the gut microbiota composition determined by weighted UniFrac distances (PERMANOVA, *p* = 0.046), but not by unweighted UniFrac distances (PERMANOVA, p = 0.51), between the patients with UC and the controls (Supplementary Fig. 4B).

Next, we examined whether gut microbiota composition was affected by mucosal inflammation in all 51 patients with UC. As MES increased, the proportion of *Bacteroides* gradually decreased (*p* = 0.061), whereas that of Enterobacteriaceae gradually increased (*p* = 0.068) (Supplementary Fig. 5A). We performed a similar analysis focusing on 10 patients who underwent biopsy during both the endoscopically active period and the remission period. The proportion of *Bacteroides* in the active period was significantly lower than that in the remission period (*p* = 0.037), whereas the proportion of Enterobacteriaceae in the active period was higher than that in the remission period (*p* = 0.027) (Supplementary Fig. 5B). The 2 α-diversity indices, namely the Chao1 index and whole-tree PD, tended to be lower in the active period than in the remission period (*p* = 0.065 and 0.065, respectively) (Supplementary Fig. 5C).

### Association between gut microbiota composition in remission period and later clinical course

**Figure 2:**
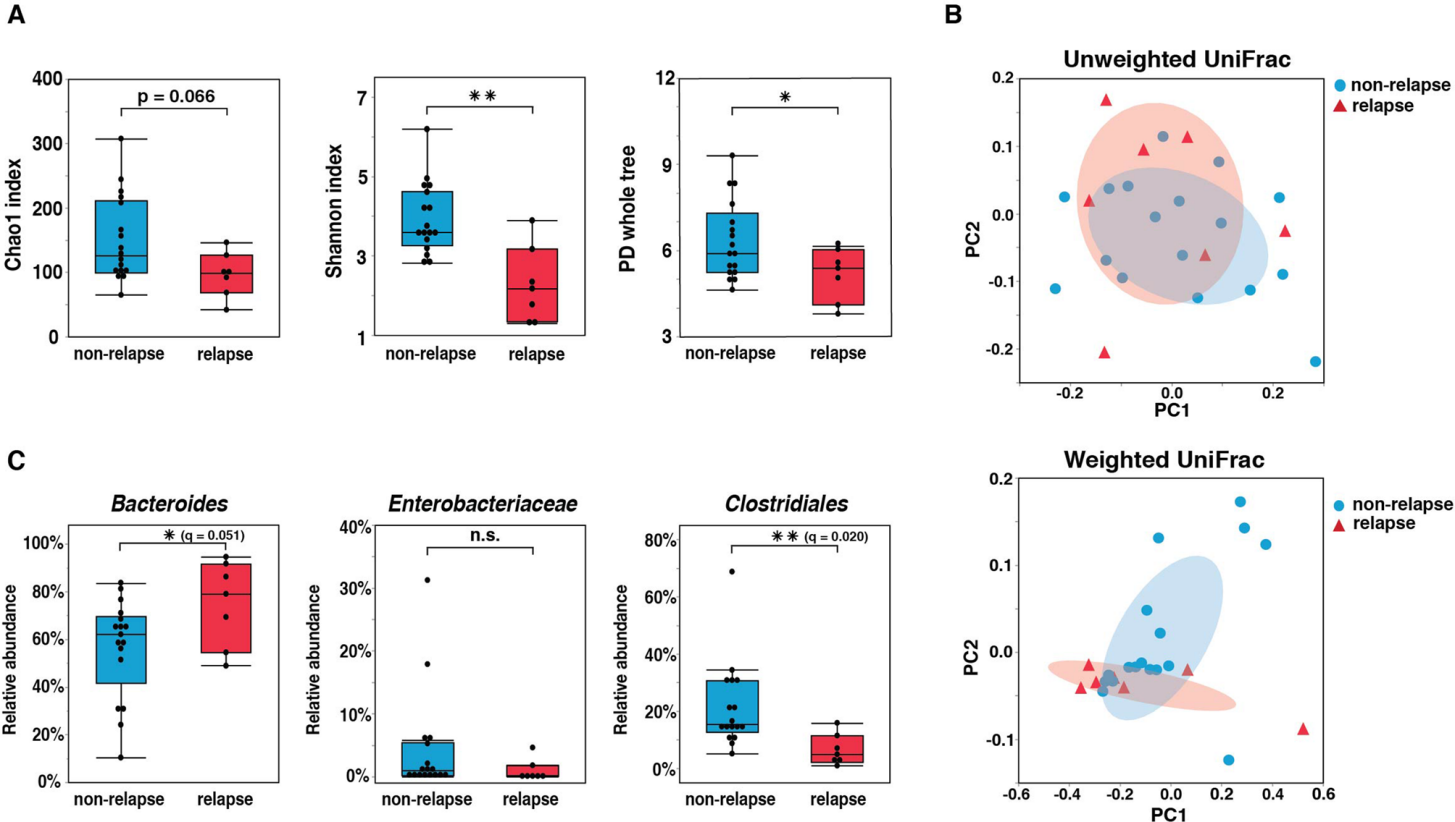
Comparison of gut microbiota composition in endoscopic remission group between non-relapse and relapse groups according to later clinical course. (**A**) Comparisons of α-diversity of gut microbiota composition between non-relapse (n = 17) and relapse (n = 7) groups as assessed by the Chao1 index, the Shannon index, and whole-tree phylogenetic diversity (PD). **(B)** Principal coordinate analysis of gut microbiota by unweighted and weighted UniFrac between non-relapse (n = 17) and relapse (n = 7) groups. Each ellipse indicates 50% probability. Unweighted (PERMANOVA, *p* < 0.0001) and weighted (PERMANOVA, *p* = 0.033) distances were significantly different. **(C)** Comparisons of taxonomic composition of microbiota including *Bacteroides,* Enterobacteriaceae, and Clostridiales between non-relapse (n = 17) and relapse (n = 7) groups. *Statistically significant difference between the 2 indicated groups (*p* < 0.05). **Statistically significant difference between the 2 indicated groups (*p* < 0.01). n.s., no significant difference between the 2 indicated groups.

We examined whether and how gut microbiota composition observed in the remission period was associated with the later clinical course. The α-diversity in the relapse group (n = 7) as measured by the Shannon index (*p* = 0.0039), whole-tree PD (*p* = 0.025), and Pielo’s evenness index (*p* = 0.0063) was significantly lower than that in the non-relapse group (n = 17), and the α-diversity in the relapse group (n = 7) as measured by the Chao1 index tended to be lower than that in the non-relapse group (*p* = 0.066) (Fig. 2A and Supplementary Fig. 2B). In contrast, there were significant differences in gut microbiota composition between the relapse and non-relapse groups as determined by the unweighted (PERMANOVA, *p* < 0.0001) and weighted (PERMANOVA, *p* = 0.033) UniFrac distances (Fig. 2B). The proportions of gut microbiota compared between the relapse and non-relapse groups by LEfSe are shown in Supplementary Fig. 6. The proportion of *Bacteroides* in the relapse group was significantly higher than that in the non-relapse group (*p* = 0.047, *q* = 0.051), and the proportion of Clostridiales in the relapse group was significantly lower than that in the non-relapse group (*p* = 0.0043, *q* = 0.020) (Fig. 2C). There was no significant difference in the proportion of Enterobacteriaceae between the 2 groups (Fig. 2C).

### Association between gut microbiota composition in active period and later clinical course

**Figure 3:**
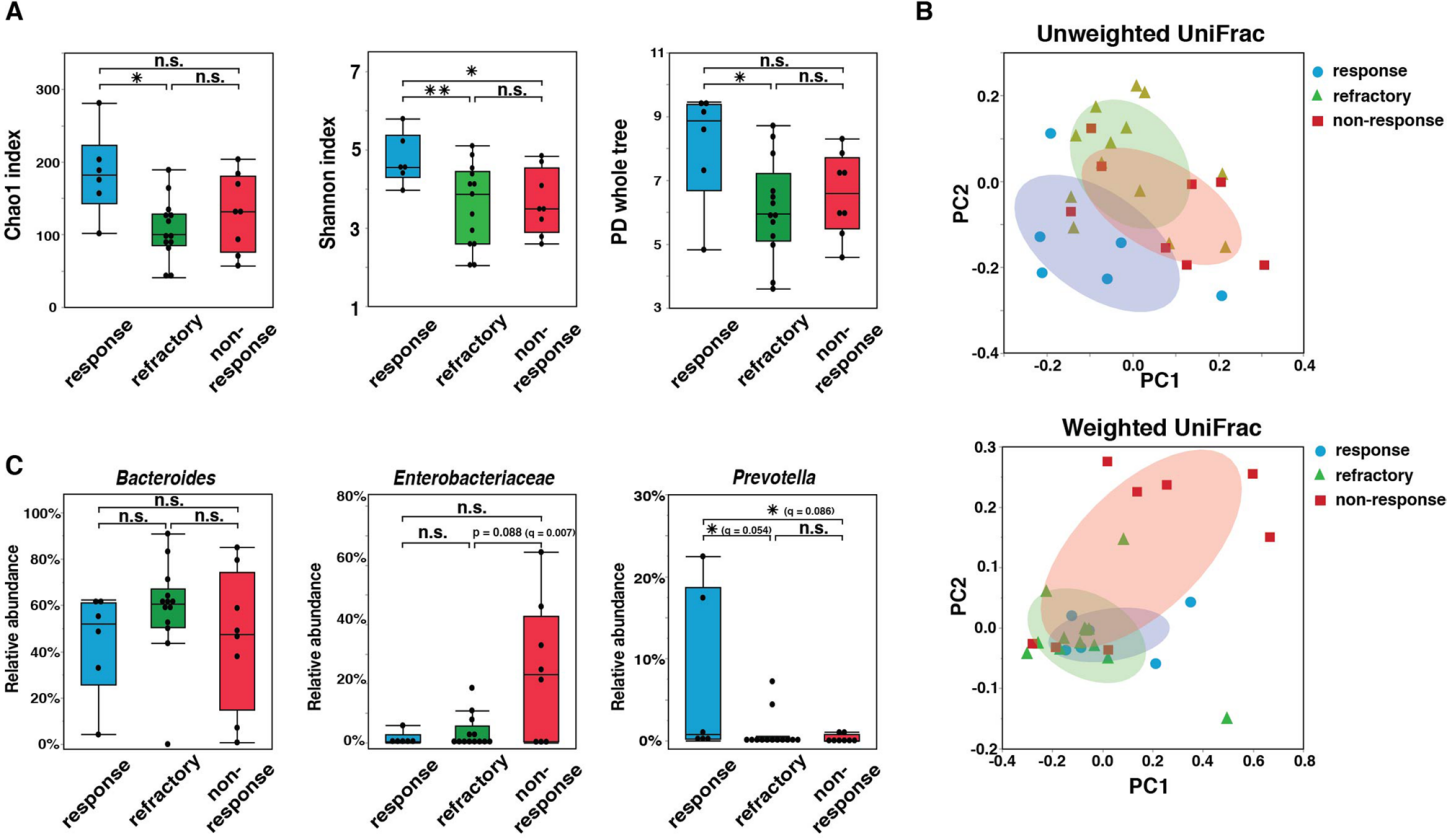
Comparison of gut microbiota composition in endoscopically active group among response, refractory, and non-response groups according to later clinical course. **(A)** Comparisons of α-diversity of gut microbiota composition among response (n = 6), refractory (n = 6), and non-response groups (n = 6) as assessed by the Chao1 index, the Shannon index, and whole-tree phylogenetic diversity (PD). **(B)** Principal coordinate analysis of gut microbiota by unweighted and weighted UniFrac among response (n = 6), refractory (n = 13), and non-response groups (n = 8). Each ellipse indicates 50% probability. **(C)** Comparison of taxonomic composition of microbiota including *Bacteroides*, Enterobacteriaceae, and *Prevotella* among response (n = 6), refractory (n = 13), and non-response groups (n = 8). *Statistically significant difference between the 2 indicated groups (p < 0.05). **Statistically significant difference between the 2 indicated groups (*p* < 0.01). n.s., no significant difference between the 2 indicated groups.

We examined whether and how gut microbiota composition in the active period was associated with the later clinical course. The α-diversity in the refractory group (n = 13) as measured by the Chao1 index (*p* = 0.011), the Shannon index (*p* = 0.025), and whole-tree PD (*p* = 0.037) were significantly lower than those in the response group. In contrast, the α-diversity in the non-response group (n = 8), as measured by Pielo’s evenness index, were significantly lower and, as measured by the Shannon index, tended to be lower than in the response group (*p* = 0.045, *p* = 0.061) (Fig. 3A and Supplementary Fig. 2C). In contrast, the PCoA of unweighted UniFrac revealed differences in gut microbiota composition among the response group, refractory group, and non-relapse group (Fig. 3B). The proportions of gut microbiota compared among the response, refractory, and non-relapse groups by LEfSe are shown in Supplementary Fig. 7. The proportion of *Prevotella* in the response group was significantly higher than that in the refractory group (*p* = 0.048, *q* = 0.054) and non-response group (*p* = 0.043, *q* = 0.086). In contrast, the proportion of Enterobacteriaceae in the non-response group tended to be higher than that in the refractory group (*p* = 0.088). There was no difference in the proportion of *Bacteroides* among the 3 groups (Fig. 3C).

### Association between gut microbiota composition and expression of Th cell subtypes

**Figure 4:**
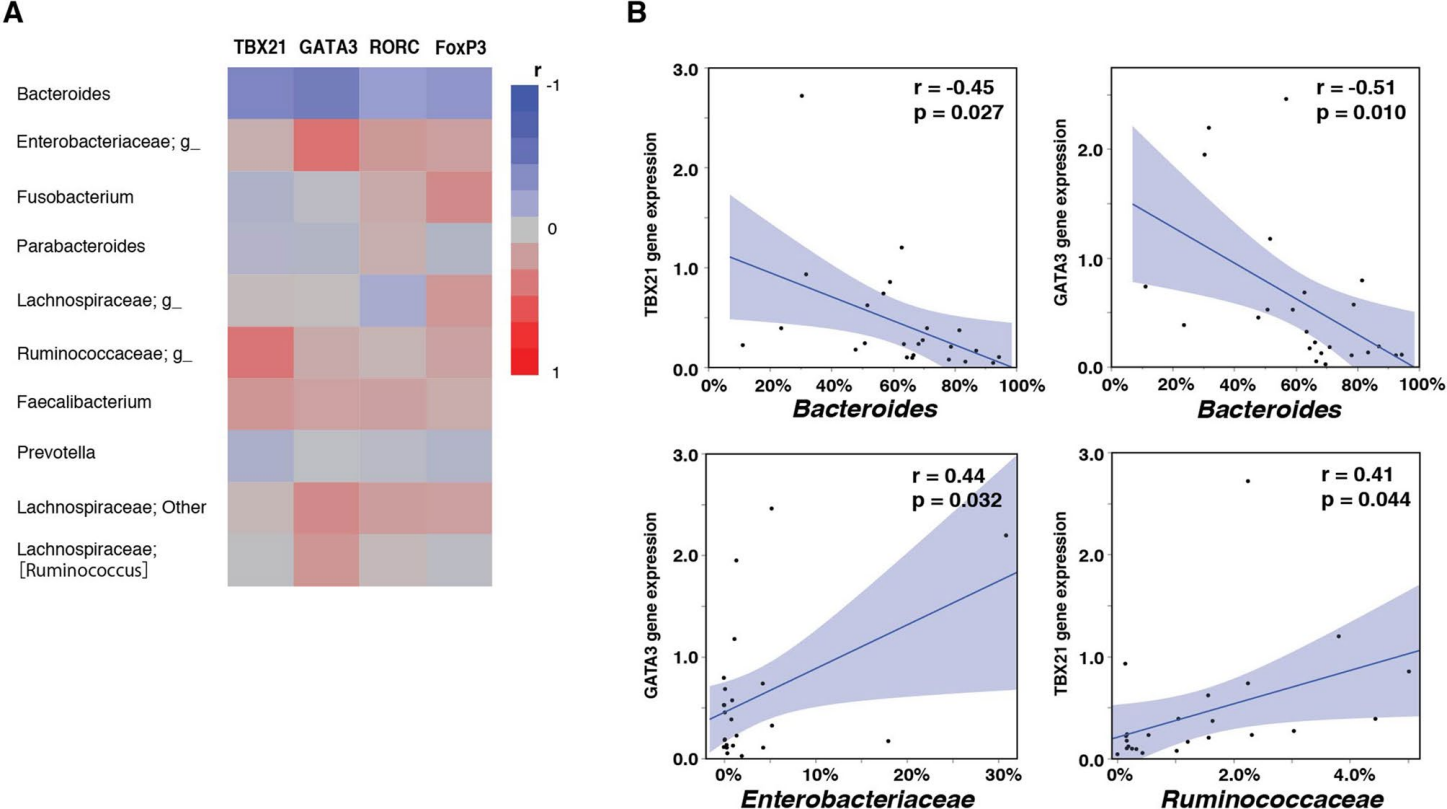
Correlation between the expression of Th-/Treg-related genes and proportion of top 10 microbes in endoscopic remission group. **(A)** Heat map of correlation between expression of T helper (Th) cell / regulatory T (Treg) cell-related genes and proportion of top 10 microbes in endoscopic remission group. **(B)** Correlation between expression of Th-/Treg-related genes and proportion of microbes. The shadow represents the 95% confidence interval of the regression line.

We next examined whether and how gut microbiota composition was associated with activation of immune cells, focusing on expression of Th cell subtypes and the top 10 microbes of highest proportion (Fig. 4A). In the endoscopic remission group (n = 24), the proportion of *Bacteroides* was inversely correlated with TBX21 (r =  − 0.45, *p* = 0.027) and GATA3 (r =  − 0.51, *p* = 0.010). In contrast, the proportion of Enterobacteriaceae was positively correlated with GATA3 (r = 0.44, *p* = 0.032) and the proportion of Ruminococcaceae was correlated with TBX21 (r = 0.41, *p* = 0.044) (Fig. 4B). In the endoscopically active group (n = 27), however, there were no significant correlations between Th cell subtypes and the top 10 microbes (Supplementary Fig. 8).

## Discussion

Several studies thus far have suggested a possible link between gut microbiota and UC. Reduced diversity together with decreased proportions of *Bacteroides* and *Faecalibacterium* and an increased proportion of *Betaproteobacteria* were reportedly observed in patients with UC^3,5^, but the association between the gut microbiota and UC is largely unknown. This study is the first demonstration that decreased diversity of gut microbiota composition is associated with the later clinical course of UC in terms of relapse events in the remission period and treatment response in the active period. In addition, we identified several microbes (*Bacteroides*, Enterobacteriaceae, Clostridiales, *Prevotella*) that were associated with the clinical course and mucosal inflammation.

In most previous studies of UC, DNA extracted from fecal samples was usually used for analysis of the gut microbiota because fecal samples are easily obtained, and extracted DNA of gut microbes can be directly amplified and sequenced against 16S rRNA. However, one concern of DNA-based 16S rRNA analysis is that it detects not only live and active microbes but also dead and dormant microbes. In contrast, RNA-based 16S rRNA analysis reflects the transcription activity of microbes, but does not detect dead or dormant microbes^8–12^. Another issue is which sample is most appropriate for 16S rRNA analysis; fecal samples or biopsied mucosal tissues. Fecal samples, which are commonly used for DNA-based 16S rRNA analysis, are easily affected by ingested food and the stool condition associated with mucosal inflammation, especially in patients with UC who have diarrhea and melena during the active phase ^6,7^. In contrast, after preparation for colonoscopy, biopsied mucosal samples are less likely to be affected by food and the stool condition^13–15^. The gut mucosal microbiota is considered to be involved in the pathophysiology of IBD in terms of immune responses because the microorganisms are directly adhered to the mucosal epithelial cells^16–18^. A previous study showed that mucosal RNA-based 16S rRNA analysis was more closely associated with the pathological condition of UC than was mucosal DNA-based 16S rRNA analysis^9^. Consistent with that finding, this study showed a significant difference in gut microbiota composition not only between feces and rectal mucosal tissues, but also between tissue DNA-based 16S rRNA and tissue RNA-based 16S rRNA. Considering the low sequencing coverage of tissue DNA-based 16S rRNA and the low quality of feces in patients with UC, tissue RNA-based 16S rRNA could be the most appropriate method for analysis of gut microbiota in patients with UC. Furthermore, whether gut microbiota of the rectal mucosa represents the colorectal microbiota remains unclear. Previous studies have shown very little difference in the microbiota composition between different colorectal sites within the same individual using DNA-based 16S.

rRNA analysis^13–15,19^. Consistently, we found no difference in the gut microbiota composition between the rectum and colon within the same individual, focusing on 14 patients who underwent 2 biopsies from the rectum and colon. Taken together, these findings suggest that the RNA-based 16S rRNA analysis using rectal mucosal tissues is reasonable to determine whether and how gut microbiota is associated with the clinical course of UC.

One of the most important findings in this study is that decreased α-diversity was associated with the later clinical course of UC in terms of relapse events during the remission period and intractability during the active period. Although the diversity of gut microbiota is reportedly lower in patients with IBD than that in healthy subjects^3^, it remains unknown how the extent of this diversity is associated with mucosal inflammation and the clinical course of UC. A previous study of pediatric patients with IBD showed that the α-diversity of fecal microbiota was inversely correlated with the clinical activity of IBD assessed by measurement of fecal calprotectin^20^. This study showed that, in the endoscopic remission group, the α-diversity in the relapse group as measured by the Shannon index and whole-tree PD was significantly lower than that in the non-relapse group. In contrast, in the endoscopically active group, the α-diversity in the refractory group as measured by the Chao1 index, the Shannon index, and whole-tree PD was significantly lower than that in the response group; furthermore, the α-diversity in the non-response group as measured by the Shannon index tended to be lower than that in the response group. These findings are consistent with the results of a previous study in which α-diversity was positively associated with treatment efficacy of fecal microbiota transplantation for patients with active UC^21^. To the best of our knowledge, this is the first study to demonstrate a relationship between α-diversity of gut microbiota and clinical course of UC (relapse and treatment response). We postulate that the α-diversity information obtained by RNA-based 16S rRNA analysis can be used to predict relapse events and treatment response in clinical practice of UC.

We have found that some microbes are associated with UC. First, the proportion of *Bacteroides* decreased as the severity of mucosal inflammation increased, not only in the cross-sectional analysis, but also in longitudinal analysis of the same patients. Importantly, the proportion of *Bacteroides* was significantly inversely associated with the gene expression of TBX21 and GATA3 in the endoscopic remission group. *Bacteroides* is reported to improve inflammation^22,23^ and is known to modulate the composition of the intestinal microbiota^24^. The findings of this study suggest that *Bacteroides* is involved in the regulation of immune responses (Th1 and Th2 cell activity) to suppress excess inflammation in the remission period. Unexpectedly, the proportion of *Bacteroides* in the non-relapse group was significantly lower than that in the relapse group during the endoscopic remission period (*p* = 0.023). The increased proportion of *Bacteroides* in the relapse group was possibly induced by compensatory mechanisms, but overgrowth of *Bacteroides* may have led to dysbiosis through reduced diversity, which might have played a role in UC relapse. Second, the proportion of Enterobacteriaceae also increased as the mucosal inflammation severity increased, not only in cross-sectional analysis, but also in longitudinal analysis of the same patients. Remarkably, a high proportion of Enterobacteriaceae in the endoscopically active period was associated with resistance to any treatments. Furthermore, during the endoscopic remission period, the proportion of Enterobacteriaceae was positively correlated with the gene expression of GATA3, which play a key role in the pathogenesis of UC^25^. Enterobacteriaceae is one of the most common inflammation-driven microbes in IBD^26,27^. An animal experiment using murine models of colitis showed that a reduction in the proportion of Enterobacteriaceae improved the severity of intestinal inflammation^28^. Taken together, these observations suggest that the Enterobacteriaceae family contains key microbes involved in the intractability that occurs during the active phase of UC. Finally, the proportion of Clostridiales was significantly higher in the non-relapse group than in the relapse group, whereas the proportion of *Prevotella* was significantly higher in the response group than in the refractory and non-response groups. Basic science studies have shown that *Clostridium* plays a role in regulation of the inflammatory response^29–31^, and that IL-10 upregulation by *Prevotella* contributes to colitis onset and progression^32^. These 4 microbes described above might play a critical role in the pathogenesis of UC, although further studies are required to clarify how they are involved.

There are several limitations to this study. With respect to medications, we excluded patients who had taken antibiotics within 3 months before the biopsies; no other medications were excluded, although there was a possibility that proton pump inhibitors affected the microbiota^33^. Second, in this study, we could not assess the influence of colonoscopy preparation on gut microbiota composition. To the best of our knowledge, there have been few studies concerning the relationship between colonoscopy preparation and gut microbiota. Considering that the majority of the gut microbiota is present in a loose outer mucus layer, the colonoscopy preparation itself might affect the composition of gut microbiota. For colon preparation, we used polyethylene glycol alone, without any surfactants or pronase enzymes that could affect the mucus layer. Thus, we presume that evaluation of the gut microbiota using mucosal samples obtained after colonoscopy preparation remains acceptable. Third, the clinical prognosis was predicted by a single test from a single colon region and the sample size was comparatively small in this study. Therefore, a large-scale prospective study with multiple tests from several colon regions is required to confirm that the key findings of our exploratory study. Fourth, not all patients underwent relapse evaluation by endoscopic examination. We acknowledge that patients without endoscopic examination might have had asymptomatic relapse, although enrolled patients with UC in remission underwent colonoscopy at intervals of 1–2 years. Finally, we could not assess whether diet, lifestyle, and metabolic diseases (e.g., diabetes and nonalcoholic steatohepatitis) had any effects on the composition of gut microbiota in this study. Diet is an important confounding factor in microbiota analysis, but we did not establish diet-related inclusion criteria during patient enrollment because it was difficult to apply such criteria to all patients. Rather than using dietary intervention, we evaluated body mass index (BMI) among the participants. Because BMI is associated with lifestyle and metabolic diseases^34^, we compared BMI among the study groups (relapse vs. non-relapse; response vs. refractory vs. non-response). Our results did not show significant differences in BMI among groups. Accordingly, we presumed that lifestyle and metabolic diseases did not strongly influence our results. Because body mass index (BMI) is associated with lifestyle and metabolic diseases^34^, we compared BMI among the study groups (relapse vs. non-relapse; response vs. refractory vs. non-response). Our results did not show significant differences in BMI among groups. Accordingly, we speculate that lifestyle and metabolic diseases did not have significant impacts on our results.

In conclusion, using RNA-based 16S rRNA analysis of rectal mucosal tissues, we have determined how the mucosa-associated gut microbiota composition is associated with the clinical course of UC. Most importantly, decreased diversity of the gut microbiota composition, as well as certain microbes, have been associated with the later clinical course of UC in terms of the relapse rate during the remission period and intractability during the active period. Of greatest clinical importance, we found that the later clinical course of UC can be predicted and treatment strategies can be determined by assessing mucosa-associated gut microbiota.

## Materials and methods

### Patient enrollment and mucosal sample collection

**Figure 5:**
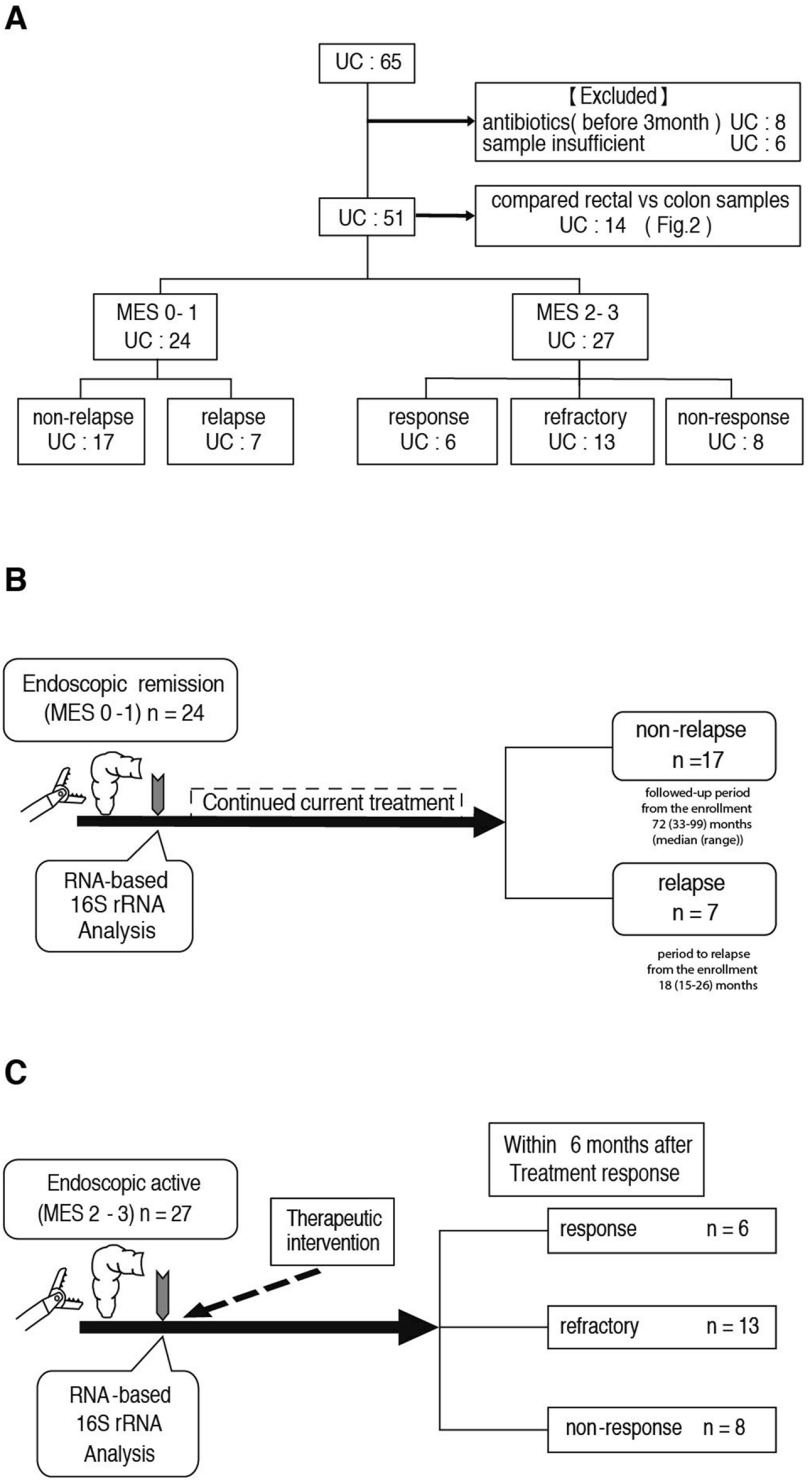
Flow chart showing the enrolled patients with ulcerative colitis (UC) and study protocols used in the present study. **(A)** Flow chart of 65 patients with UC initially enrolled in this study. Fourteen patients were excluded, and we analyzed a total of 51 patients with UC. They were classified into 2 groups: those with a Mayo endoscopic score (MES) of 0/1 (endoscopic remission group, n = 24) and those with an MES of 2/3 (endoscopically active group, n = 27). **(B)** Study protocol of the endoscopic remission group. After the biopsy samples for RNA-based 16S rRNA analysis had been obtained from the patients with an MES of 0/1 (n = 24), they were classified into 2 groups according to their later clinical course: non-relapse (n = 17) and relapse (n = 7). **(C)** Study protocol of the endoscopically active group. After the biopsy samples for RNA-based 16S rRNA analysis had been obtained from the patients with an MES of 2/3 (n = 27), they were classified into 3 groups according to their later clinical course: response (n = 6), refractory (n = 13), and non-response (n = 8).

Inclusion criteria for patients with UC were as follows: diagnosis with UC before enrollment, planned colonoscopy for UC, age > 20 years, and provision of written informed consent. Exclusion criteria for patients with UC were either of the following: bleeding tendency and determination of poor suitability by the attending physician. In contrast, inclusion criteria for control subjects were as follows: planned colonoscopy for the examination or treatment of colon polyps, age > 20 years, and provision of written informed consent. Exclusion criteria for control subjects were any of the following: any intestinal inflammation, bleeding tendency, and determination of poor suitability by the attending physician. From June 2012 to December 2017, 65 consecutive patients with UC and 13 control subjects who met the inclusion criteria were enrolled in this longitudinal observational study at Kyushu University Hospital. Six of these 13 control subjects were enrolled to study the detection yield of the gut microbiota based on combinations of samples used and methods applied, whereas the remaining 7 control subjects were enrolled for comparison with the patients with UC. Among the 65 patients with UC, we excluded 10 patients who had taken antibiotics within the prior 3 months before we obtained the biopsy samples and 4 patients whose samples were insufficient for analysis. Thus, in total, 51 patients with UC were analyzed in this study. Based on their colonoscopy findings, the 51 patients comprised 10, 14, 13, and 14 patients with a Mayo endoscopic score (MES) of 0, 1, 2, and 3, respectively. The endoscopy score was assessed in a blinded manner in the present study; the endoscopists differed from the attending physicians who were in charge of patient treatment. The patients with UC were classified into an endoscopic remission group (MES of 0/1, n = 24) and an endoscopically active group (MES of 2/3, n = 27) (Fig. 5A). The biopsied samples were obtained from the rectum and, in 14 patients with UC, the biopsied samples were obtained from the colon in addition to the rectum when the patients gave informed consent to obtain colonic samples. The samples, thus obtained, were immersed in RNAlater (Ambion, Inc., Austin, TX, USA) immediately after collection, transferred to a freezer (− 30 °C), and stored until further use.

### Clinical information

An MES of 0/1 was defined as endoscopic remission, while an MES of 2/3 was defined as endoscopically active. In analysis of the remission group (Fig. 5B), non-relapse was defined as the maintenance of remission (MES of ≤ 1 or a Partial Mayo Scoring Index of ≤ 4); relapse was defined as a change to an MES of ≥ 2 or a Partial Mayo Scoring Index of > 4. Patients in the endoscopic remission group had been followed-up from the time of enrollment until August 2020 (non-relapse group: 33–99 months) or from enrollment to relapse (relapse group: 15–26 months). No patients were enrolled immediately after an induction treatment; all patients with MES0/1 had maintained remission for more than 1 year. The endoscopic remission group (MES of 0/1, n = 24) was further classified into 2 groups based on the patients’ clinical course : the non-relapse group (n = 17) and the relapse group (n = 7) (Fig. 5B). The endoscopically active group (MES of 2/3, n = 27) was also further classified into 3 groups based on the 6-month therapeutic course after the biopsy were obtained : the response group (n = 6), refractory group (n = 13), and non-response group (n = 8) (Fig. 5C). The patients in the response group achieved remission by conventional treatments (5-aminosalicylic acid, azathioprine, mercaptopurine, apheresis, and prednisolone). The patients in the refractory group included those with prednisolone dependence/resistance and those who had achieved remission by step-up treatment of anti-tumor necrosis factor biologics or tacrolimus, but not by conventional treatments. The patients in the non-response group included those who had undergone surgical treatment and those who had not achieved remission by any medical treatments. A specific sample size was not established because this study was exploratory in nature.

### Extraction of RNA, reverse transcription, and 16S rRNA amplicon sequencing

Total RNA was extracted from whole biopsy tissues using TRIzol reagent (Invitrogen, Carlsbad, CA, USA), and its concentration and purity were measured at 260 and 280 nm using a NanoDrop ND-1000 UV/Vis spectrophotometer (NanoDrop Technologies, Thermo Fisher Scientific, Waltham, MA, USA)^35^. The V1–V2 region of 16S rRNA was amplified from cDNA using the universal primers Tru 27F and Tru 354R^36^ (Supplementary Table 1). Reverse transcription was performed using the Tru 354R primer and a PrimeScript II 1st Strand cDNA Synthesis Kit (Takara Bio, Shiga, Japan). The reverse transcription conditions were as follows: 42 °C for 45 min followed by 70 °C for 15 min. Polymerase chain reaction (PCR) was performed by Takara Ex Taq HS DNA polymerase (Takara Bio). The PCR conditions were as follows: 94 °C for 3 min; 24 cycles at 94 °C for 30 s, 50 °C for 30 s, and 72 °C for 30 s; and finally 72 °C for 10 min. A barcode sequence tag was added to each amplicon by a second PCR. The conditions of this second PCR procedure were as follows: 94 °C for 3 min; 10 cycles at 94 °C for 30 s, 60 °C for 30 s, and 72 °C for 30 s; and finally 72 °C for 10 min. A pool of equal amounts (20 ng) of amplicons from each sample was purified using an ethanol precipitation method. The amplicon mixture was then subjected to paired-end sequencing by an Illumina MiSeq System (Illumina, Inc., San Diego, CA, USA). The obtained sequences were processed using the UPARSE pipeline in USEARCH^37^. First, the pairs of sequence reads were merged using the -fastq-mergepairs command. After quality filtering, length trimming, dereplication, and discarding of singletons, the merged sequences were clustered into 1339 operational taxonomic units (OTUs), each representing > 96% identity, using the UPARSE-OTU algorithm. In total, 1.20 × 10^6^ reads were retained (median, 9.36 × 10^3^ reads; range, 7.85 × 10^3^ to 1.19 × 10^4^).

The taxonomy of each OTU was assigned using the Greengenes reference sequence database (gg_13_5)^38^ and the UCLUST algorithm (assign_taxonomy.py) in QIIME^39^. The taxonomic composition of each biopsy sample was determined based on the OTU table using the QIIME summarize_taxa_througy_plots.py command. Sequence data were deposited in DDBJ under accession numbers DRA 011218 and BioProject PRJDB 10871.

### α-Diversity analysis

The α-diversity of gut microbiota composition was calculated using 3 standard indices: the Chao1 index, the Shannon index, and whole-tree phylogenetic diversity (PD), with the OTU table rarefied to 5000 sequences per sample with 10 random interactions using the QIIME alpha_rarefaction.py command. Pielo’s evenness index was calculated by using Shannon index.

### β-Diversity analysis

The pairwise-weighted UniFrac distance of gut microbiota composition between samples was calculated based on the OTU table rarefied to 5000 sequences per sample using β_diversity_through_plot.py in QIIME. Differences in community components were statistically analyzed based on the weighted and unweighted UniFrac metric using the “Adonis” function with 999 permutations in the QIIME pipeline.

### Quantitative real-time PCR

The expression of mRNAs encoding transcription factors was determined by quantitative PCR using TaqMan Gene Expression Assay Reagent (Applied Biosystems, Foster City, CA, USA). The targets were transcription factors related to T helper 1 (Th1) cells (TBX21), Th2 cells (GATA3), Th17 cells (retinoic acid receptor-related orphan receptor C [RORC]), and regulatory T cells (forkhead box P3 [FoxP3]); these were assessed using the primer–probe sets listed in Supplementary Table 1. The cDNA equivalent of 10 ng of the RNA, specific primers, and TaqMan Universal PCR Master Mix, No AmpErase UNG (Applied Biosystems) were added to each well; the volume was adjusted to 20 μL; and thermal cycling reactions were performed using a 7500 Real-Time PCR System (Applied Biosystems). The amplification protocol consisted of initial denaturation at 95 °C for 10 min, followed by 40 cycles of denaturation at 95 °C for 15 s and annealing and extension at 60 °C for 60 s. The internal control consisted of primers amplifying a sequence of glyceraldehyde-3-phosphate dehydrogenase. The comparative CT (2^−ΔΔCT^) method was used for subsequent statistical analysis^35,40^.

### Statistical analysis

All numerical data are expressed as median and interquartile range unless otherwise stated. The boxes represent the 25th to 75th percentiles, and the whiskers indicate the minimum and maximum values excluding outliers. The Shapiro–Wilk test for normality was used to divide continuous data according to a normal or non-normal distribution. Student’s *t* test was used to compare normally distributed data between 2 groups, and the Mann–Whitney U test and Wilcoxon matched-pairs signed rank test were used to compare non-normally distributed data between 2 groups. The Tukey–Kramer test for normally distributed data and the nonparametric Wilcoxon rank sum test for non-normally distributed data were used to determine statistical significance among more than 2 groups. Proportions of gut microbiota were first evaluated by using Linear discriminant analysis effect size^41^ (LEfSe) assessment; microbiota regarded as biologically relevant^3,5^ were assessed by Benjamini–Hochberg-corrected analysis with *q* values. Trend analysis was performed using a linear trend test. A linear regression analysis was used to assess whether dependent variables (TBX21, GATA3, RORC, and FoxP3) were related to the top 10 microbes with high composition. β-Diversity was estimated using the UniFrac metric to calculate the distances of the gut microbiota composition between the samples and was visualized by principal coordinate analysis (PCoA) and statistically analyzed using permutational multivariate analysis of variance (PERMANOVA)^19^. A p value of < 0.05 was considered statistically significant. All calculations, statistical analyses, and scatterplot drawings were performed using the JMP Pro software program, version 14.0.0 (SAS Institute, Inc., Cary, NC, USA).

### Ethical considerations

The aim of this study was explained to each patient during the interview before endoscopy. All patients were informed of the potential risk of bleeding from the biopsy sites, although the risk was considered to be low. Only patients who gave their written informed consent were included. This study was reviewed and approved by Kyushu University Certified Institutional Review Board for Clinical Trials and conducted in accordance with the ethical principles of the Declaration of Helsinki. The study has been registered with Kyushu University Hospital under the identification number 30–472.

## Supplementary Information

Supplementary Information 1.

Supplementary Information 2.

Supplementary Information 3.

Supplementary Information 4.

Supplementary Information 5.

Supplementary Information 6.

Supplementary Information 7.

Supplementary Information 8.

Supplementary Information 9.

Supplementary Information 10.
