# Supplementary material for: Mucosa-associated gut microbiota reflects clinical course of ulcerative colitis
Source: Sci Rep. 2021 Jul 2;11:13743. doi: 10.1038/s41598-021-92870-0 (PMC8253849; doi:10.1038/s41598-021-92870-0)
Supplement: Supplementary file 1 — Supplementary Information 1. [file 41598_2021_92870_MOESM1_ESM.docx]

**Mucosa-associated gut microbiota reflects clinical course of ulcerative colitis**

Yuichiro Nishihara, MD, Haruei Ogino, MD, PhD, Masaru Tanaka, PhD,

Eikichi Ihara, MD, PhD, Keita Fukaura, MD, PhD, Kei Nishioka, MD,

Takatoshi Chinen, MD, PhD, Yoshimasa Tanaka, MD, PhD,

Jiro Nakayama, PhD, Dongchon Kang, MD, PhD, and Yoshihiro Ogawa, MD, PhD

**Supplementary Methods**

**Extraction of DNA and RNA from fecal and rectal biopsy specimens followed by 16S rRNA amplicon sequencing**

Six control subjects were enrolled to study the detection yield of the gut microbiota based on the combinations of samples used and methods applied. Each patient collected a fecal sample prior to preparing for colonoscopy. The fecal and rectal biopsy samples were immersed in RNAlater (Ambion, Inc., Austin, TX, USA) immediately after collection, transferred to a freezer (−30°C), and stored until further use. The fecal and rectal biopsy specimens were homogenized, and DNA and RNA were extracted using an AllPrep PowerFecal DNA/RNA Kit and AllPrep DNA/RNA Mini Kit (QIAGEN, Hilden, Germany). 16S rRNA amplicon sequencing and analysis were performed using the above-described method. The merged sequences were clustered into 1214 operational taxonomic units (OTUs), each representing >96% identity, using the UPARSE-OTU algorithm. In total, 9.55 × 10^5^ reads were retained from 24 samples (median, 4.17 × 10^4^ reads; range, 3.28 × 10^3^ to 4.67 × 10^4^).

**Supplementary Table 1. Primers used for amplicon preparation and qPCR**

**(A)** Base sequence of the amplicon primer. **(B)** List of genes analyzed by qPCR (*TBX21*, *GATA3*, *RORC*, and *FOXP3*).

**Supplementary Figure 1. Comparisons of gut microbiota composition analyzed by 16S rRNA based on combination of samples (feces vs. tissues) and methods (DNA-based vs. RNA-based) in 6 control subjects**

**(A)** Number of samples that achieved the indicated sequencing depth analyzed by 16Sr RNA based on 4 different conditions: F-DNA, F-RNA, T-DNA, and T-RNA. **(B)** Rarefaction curves of observed operational taxonomic units (OTUs). The bar represents the standard error. **(C)** Comparison of proportion of unclassified bacteria between T-DNA and T-RNA. **(D)** Principal coordinate analysis of gut microbiota by unweighted and weighted UniFrac analyzed by 4 different conditions: F-DNA, F-RNA, T-DNA, and T-RNA in 6 control subjects. The color of the symbol indicates the 4 different conditions, and the shape of the symbol indicates one patient. Unweighted (PERMANOVA, p < 0.0001) and weighted (PERMANOVA, p = 0.0001) distances were significantly different between feces and tissue, and unweighted (PERMANOVA, p = 0.0001) distances were significantly different between T-DNA and T-RNA. F-DNA, feces DNA-based 16S rRNA; F-RNA, feces RNA-based 16S rRNA; T-DNA, tissue DNA-based 16S rRNA; T-RNA, tissue RNA-based 16S rRNA.

**Supplementary Figure 2. Comparison of Pielo’s evenness index among each groups**

**(A)** Comparisons of α-diversity of gut microbiota composition between rectum and colon as assessed by the Pielo’s evenness index. **(B)** Comparisons of α-diversity of gut microbiota composition between non-relapse (n = 17) and relapse (n = 7) groups as assessed by the Pielo’s evenness index. **(C)** Comparisons of α-diversity of gut microbiota composition among response (n = 6), refractory (n = 6), and non-response groups (n = 6) as assessed by the Pielo’s evenness index. **(D)** Comparisons of α-diversity of gut microbiota composition between patients with UC (n = 51) and control subjects (n = 7) as assessed by the Pielo’s evenness index. **(E)** Comparisons of α-diversity of gut microbiota composition between patients with UC (MES0/1; n = 24, MES2/3; n = 27) and control subjects (n = 7) as assessed by the Pielo’s evenness index. **(F)** Changes in α-diversity of gut microbiota composition between the endoscopic remission period (MES 0/1) and active period (MES 2/3) as assessed by the Pielo’s evenness index in 10 patients with UC who underwent biopsies in 2 different periods. Statistically significant difference between the 2 indicated groups (*p < 0.05, ** p < 0.01).

**Supplementary Figure 3. Intraindividual and interindividual comparisons of gut microbiota composition**

Intraindividual (rectum vs. colon) and interindividual (rectum vs. rectum) comparisons of gut microbiota composition. **Statistically significant difference between the 2 indicated groups (p < 0.01).

**Supplementary Figure 4. Comparison of gut microbiota composition between patients with ulcerative colitis (UC) and control subjects**

**(A)** Comparisons of α-diversity of gut microbiota composition between patients with UC (n = 51) and control subjects (n = 7) as assessed by the Chao1 index, the Shannon index, and whole-tree phylogenetic diversity (PD). **(B)** Principal coordinate analysis of gut microbiota by unweighted and weighted UniFrac between patients with UC (n = 51) and control subjects (n = 7). Each ellipse indicates 50% probability. Weighted distances were significantly different between patients with UC and control subjects (PERMANOVA, p = 0.046). *Statistically significant difference between the 2 indicated groups (p < 0.05).

**Supplementary Figure 5. Changes in gut microbiota composition based on endoscopic severity of mucosal inflammation**

**(A)** Changes in the proportion of *Bacteroides* and Enterobacteriaceae based on the endoscopic severity of mucosal inflammation by the Mayo endoscopic score (MES) in 51 patients with ulcerative colitis (UC). **(B)** Changes in the proportion of *Bacteroides* and Enterobacteriaceae between the endoscopic remission period (MES 0/1) and active period (MES 2/3) in the 10 patients with UC who underwent biopsies in 2 different periods. **(C)** Changes in α-diversity of gut microbiota composition between the endoscopic remission period (MES 0/1) and active period (MES 2/3) as assessed by the Chao1 index, the Shannon index, and whole-tree phylogenetic diversity (PD) in 10 patients with UC who underwent biopsies in 2 different periods.

**Supplementary Figure 6. Comparison of gut microbiota composition in the endoscopic remission group between non-relapse and relapse groups by Linear discriminant analysis Effect Size (LEfSe) assessment.**

**Supplementary Figure 7 Comparison of gut microbiota composition in the endoscopically active group among response, refractory, and non-response groups by Linear discriminant analysis Effect Size (LEfSe) assessment.**

**Supplementary Figure 8. Correlation between expression of Th-/Treg-related genes and proportion of top 10 microbes in endoscopically active group**

Heat map of correlation between expression of Th-/Treg-related genes and proportion of top 10 microbes in endoscopically active group (n = 27).
