# Supplementary material for: Mucosa-associated gut microbiota reflects clinical course of ulcerative colitis
Source: Sci Rep. 2021 Jul 2;11:13743. doi: 10.1038/s41598-021-92870-0 (PMC8253849; doi:10.1038/s41598-021-92870-0)

Mucosa-associated gut microbiota reflects clinical course of ulcerative colitis

Yuichiro Nishihara, MD, Haruei Ogino, MD, PhD, Masaru Tanaka, PhD, Eikichi Ihara, MD, PhD,
Keita Fukaura, MD, PhD, Kei Nishioka, MD, Takatoshi Chinen, MD, PhD, Yoshimasa Tanaka, MD, PhD,
Jiro Nakayama, PhD, Dongchon Kang, MD, PhD, and Yoshihiro Ogawa, MD, PhD

Supplementary Figure 8

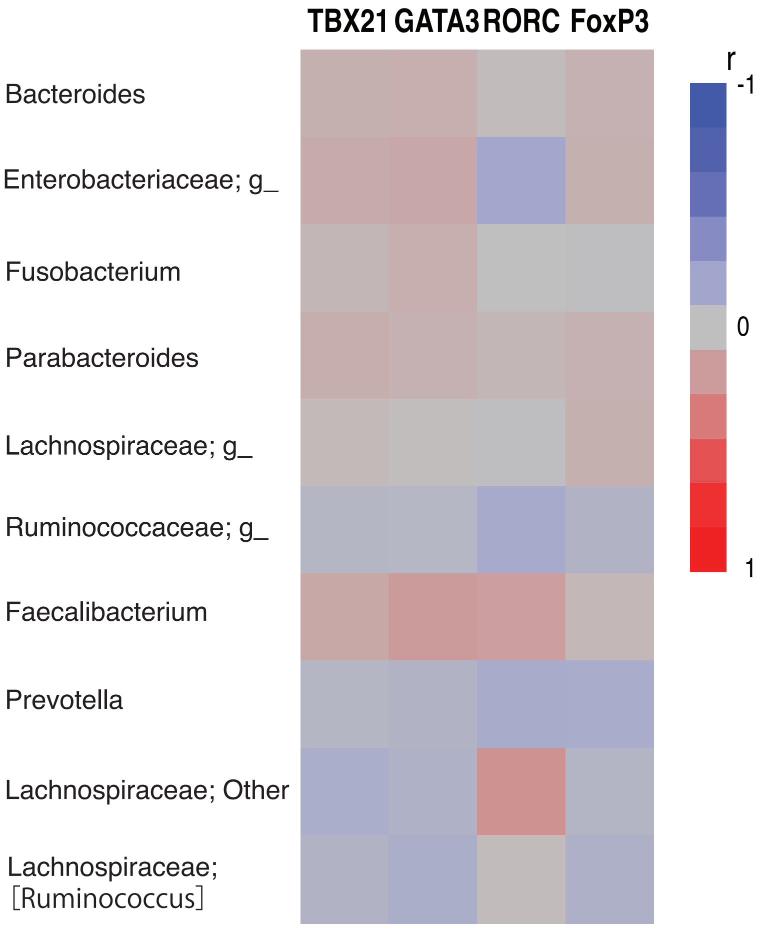

Supplement: Supplementary file 9 — Supplementary Information 9. [file 41598_2021_92870_MOESM9_ESM.pdf]
