# Supplementary material for: Mucosa-associated gut microbiota reflects clinical course of ulcerative colitis
Source: Sci Rep. 2021 Jul 2;11:13743. doi: 10.1038/s41598-021-92870-0 (PMC8253849; doi:10.1038/s41598-021-92870-0)
Supplement: Supplementary file 10 — Supplementary Information 10. [file 41598_2021_92870_MOESM10_ESM.docx]

**Mucosa-associated gut microbiota reflects clinical course of ulcerative colitis**

Yuichiro Nishihara, MD, Haruei Ogino, MD, PhD, Masaru Tanaka, PhD,

Eikichi Ihara, MD, PhD, Keita Fukaura, MD, PhD, Kei Nishioka, MD,

Takatoshi Chinen, MD, PhD, Yoshimasa Tanaka, MD, PhD,

Jiro Nakayama, PhD, Dongchon Kang, MD, PhD, and Yoshihiro Ogawa, MD, PhD

**Supplementary Table 1**

A

Base Sequence of the amplicon primer (V1-V2 region of the 16S rRNA)

|  | ***Base Sequence*** |
| --- | --- |
| **Tru 27F**  **Tru 354R** | 5’ – CCCTCTTCCGATCTCTGAGRGTTTGATYMGGCTCAG – 3’ |
|  | 5’ – TGCTCTTCCGATCTGACCTGCCTCCCCTAGGAGT – 3’ |

B

List of genes analyzed in samples by qPCR

|  | ***Encoding Genes*** | **ABI assay number** ^a^ |
| --- | --- | --- |
| **Transcription factor** | *TBX21* (T-bet) | Hs00203436_m1 |
|  | *GATA3* (GATA3) | Hs00231122_m1 |
|  | *RORC* (RORC) | Hs00172860_m1 |
|  | *FOXP3* (Foxp3) | Hs00203958_m1 |
|  |  |  |

Gene symbols are italicized. ^a^ Applied Biosystems gene expression assay number. RORC, retinoic acid receptor-related orphan receptor C; FoxP, forkhead box P; qPCR, quantitative real-time polymerase chain reaction.
